# Drying Shrinkage of Concrete Containing Calcium Stearate, (Ca(C_18_H_35_O_2_)_2_), with Ordinary Portland Cement (OPC) as a Binder: Experimental and Modelling Studies

**DOI:** 10.3390/molecules25214880

**Published:** 2020-10-22

**Authors:** Agus Maryoto, Rachmad Setijadi, Arnie Widyaningrum, Sugeng Waluyo

**Affiliations:** 1 Department of Civil Engineering, Universitas Jenderal Soedirman, Jl. Mayjend Sungkono KM 5, Blater, Purbalingga, Central Java 53371, Indonesia; arnie.widyaningrum@unsoed.ac.id; 2 Department of Geology Engineering, Universitas Jenderal Soedirman, Jl. Mayjend Sungkono KM 5, Blater, Purbalingga, Central Java 53371, Indonesia; rsetijadi_ianov@yahoo.co.id; 3 Department of Industrial Engineering, Universitas Jenderal Soedirman, Jl. Mayjend Sungkono KM 5, Blater, Purbalingga, Central Java 53371, Indonesia; sugeng.waluyo@unsoed.ac.id

**Keywords:** calcium stearate, shrinkage, experiment, shrinkage model

## Abstract

This work investigates the effect of calcium stearate (Ca(C_18_H_35_O_2_)_2_) on concrete shrinkage behaviors by using experimental testing. The test specimens are cubes with each dimension given as 100 × 100 × 285 mm for shrinkage tests and cylinders with 150 mm diameter and 300 mm height for compressive strength tests. The calcium stearate with fractions of 0, 0.1, 0.2, and 0.3% from the weight of cement are used in the tests. The results showed that the shrinkage occurred in amounts of 0.079, 0.062, 0.065, and 0.060 mm for the specimens containing calcium stearate of 0, 0.1, 0.2, and 0.3%, respectively. Moreover, we also perform shrinkage modelling to explore a possibility to incorporate the calcium stearate fraction into the standard concrete shrinkage model. There are three well-known shrinkage models used here, i.e., the Sakata, the Japan Standard and the Bazant-Baweja models, where only the latter one is capable to capture our experimental results very well for different fractions of calcium stearate.

## 1. Introduction

Concrete is artificial rock composed from cement, crushed stone, sand, water, and some additional materials with minor fraction. The hydration reaction process between cement and water takes place continuously over time. Although generally the compressive strength of concrete at 28 days is used, in fact the strength beyond 28 days continues to develop. During the hardening of the concrete, some of the water from the concrete evaporates and affects the reduction of the hard concrete volume. This reduction in concrete volume over time is called concrete shrinkage.

The shrinkage can occur before the initial setting time of concrete production and is often called plastic shrinkage. On the other hand, for the shrinkage after the initial setting time, one calls it drying shrinkage. If the initial setting time is exceeded, the concrete will enter the hardening process, where the water in concrete capillaries will evaporate and induce shrinkage. The process will generate induced tensile stress field in concrete. Hence, cracks will be formed due to low tensile strength capacity of concrete.

Moreover, many factors can affect the level of shrinkage, which include environmental conditions, the exposed surface area of the concrete, the amount of concrete constituent material, the physical properties of the concrete constituent aggregate, and the water-cement ratio [1]. Some added admixture or additive such as water entraining agent [2], slag [3], fly ash [4,5], biomass ashes [6], waterproof material [7], and also the type of binder used, such as ordinary Portland cement (OPC), Portland composite cement (PCC) and Portland pozzolan cement (PPC), have effects on the shrinkage of the concrete as well. Additionally, fly ash usage in the conrete usually reduces the drying shrinkage both in normal concrete and in self-compacting concrete [8,9,10]. On the other hand, water-resistant materials are commonly used to cover concrete surface to reduce the number of capillaries present inside concrete in order to reduce the water-cement ratio [11]. Concrete workability with a low water-cement ratio is maintained when the concrete uses superplasticizers.

As we mentioned before, any existing cracks due to shrinkage can affect the carrying capacity of reinforced concrete. Tiny cracks that arise during plastic shrinkage will propagate during the concrete hardening phase and reduce the bond between steel bars and concrete. As a result, reinforced concrete cannot work comprehensively in supporting the external load. In addition, if reinforced concrete is located in a corrosive environment, infiltration of chloride ions, sulfates or other corrosive material can occur through this very small crack width. The reinforcing steel bar will corrode and cause the spalling of a concrete cover [12]. Then, the corrosion will attack the steel bar faster, because the steel bar has no concrete cover. Finally, the carrying capacity of reinforced concrete is reduced significantly due to corrosion of the steel bar. The service life of reinforced concrete structures decreases. To repair the decreasing capacity load of the beam, it can be strengthened by using wire rope [13].

The effect of calcium stearate in the concrete has been studied intensively by some researchers, e.g., to reduce the permeability [14], to act as an accelerator and reduce the corrosion rate [15], to improve absorption in the foamed concrete [16], and also to reduce the quantity of macroscopic sale pore sized 10–100 µm [17]. The reduction of water absorption can also be applied in the concrete by adding the superplasticiser as a water reducer [18]. The application of superplasticizer in concrete automatically decreases the free water in concrete. As a result, the capillaries in concrete will go down due to evaporation of free water in fresh concrete. Other comprehensive researches conducted by Maryoto et al. [19,20,21] have also found that the use of calcium stearate in concrete reduces compressive strength, corrosion attacks, chloride ion infiltration, and water absorption. The reduction of corrosion attacks can also be avoided by applying an inhibitor in the reinforced concrete [22]. According to their hypothesis, calcium stearate reacts with cement and water to form hydrophobic compounds and to cover the surface of concrete capillaries in the form of wax-like compounds. During the hydration process, the wax-like constituent is left inside the capillaries, so the capillary diameter becomes smaller with the addition of calcium stearate. However, the advantage of the physical properties of concrete with calcium stearate is not useful if cracks occur in concrete structures. Therefore, the objective of this research is to investigate the shrinkage of concrete containing calcium stearate. Shrinkage is a very important property in concrete in terms of concrete durability. A significant amount of shrinkage in concrete can produce cracks allowing material infiltrations and producing corrosion in some condition. For example, corrosive ion such as chloride has been known to be responsible for attacking the surface of steel bar in concrete through cracks.

Additionally, we perform concrete shrinkage modelling to incorporate the influence of calcium stearate into the standard model of concrete shrinkage models. To the best of our knowledge, there are still no standard shrinkage models related directly to the influence of calcium stearate. However, according to our hypothesis, the calcium stearate in concrete may strongly contribute to the concrete actual water content before shrinkage occurs. Hence, we may use shrinkage models, which depend explicitly on water content as a variable. For this reason, we choose in this work the Sakata [23], the Japan Standard [24] and the Bazant-Baweja [25] models.

## 2. Materials and Methods

**Figure 1:**
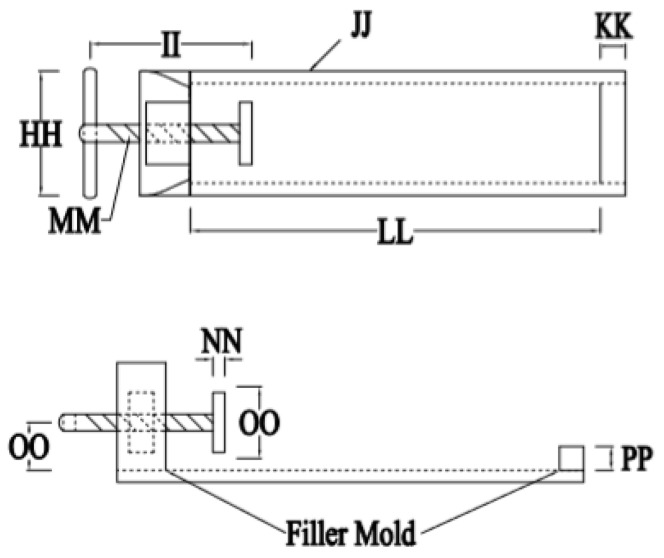
Device for shrinkage test.

**Figure 2:**
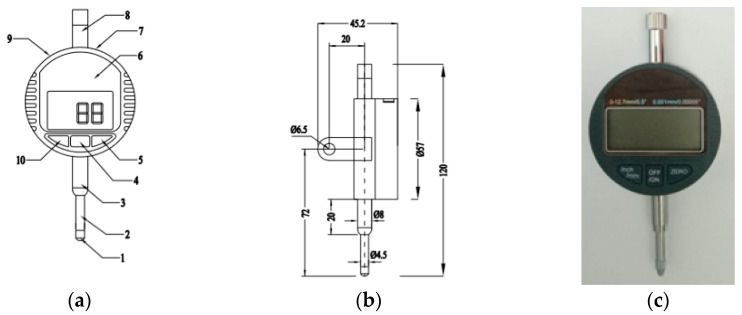
Digital micro dial gauge indicator: (**a**) top view, (**b**) side view, (**c**) top view of real equipment.

Our test was done in our own laboratory with controlled air temperature and humidity. The materials used included OPC type cement, crushed stone, sand, water, and calcium stearate. The equipment used consisted of a concrete mixer, slump test equipment, oven, sieve analyzer, balance, cylinder molding diameter 150 mm and height 300 mm, tamping rod, beam molding for shrinkage specimens 100 × 100 × 285 mm, and a set of devices to conduct the shrinkage. The test set-up is shown in Figure 1 according to [26] where the digital micro dial gauge indicator is shown separately in Figure 2.

We define some dimensional variables in Figure 1 as HH = 100 mm, II = 130 mm, JJ = 8 cm channel, KK = 20 mm, LL = 330 mm, MM = 14 mm, NN = 20 mm, OO = 50 mm, and PP = 20 mm.

In Figure 2a, the numbering on the gauge indicator is given from 1 till 9 as the contact point, the spindle, the mounting diameter, the on/off switch, the zero setting, the LCD display, the data output interface, the anti-dust cap, the battery compartment, and the inch/mm switch, respectively.

Furthermore, the digital micro indicator can be set on a zero setting at any point. The on/off button means multiple functions, i.e., data hold function, fast display, and tracing of maximum or minimum value. The metric/inch system can be interchanged at any position. When the on/off switch is pressed, 000 mm will appear on the display. This means that the indicator is now ready to be used for measurements.

### 2.1. Preliminary Testing

Preliminary testing was done to find out the initial data of constituent material of concrete in order to achieve concrete mix designs for 20 MPa concrete quality. The preliminary tests on sand and crushed stone consist of the fineness modulus, fill weight, specific gravity, and clay content (Table 1). In addition, abrasion tests are also carried out on the crushed stones. The cement is tested for its physical and chemical properties. The physical properties and chemical content of cement are shown in Table 2 and Table 3. The preliminary test data are used to design the concrete mix proportions as listed in Table 4.

In the Table 2 and Table 3 SNI is the Indonesian National Standard. The physical and chemical cement test results indicate that ordinary Portland cement meets the requirements that must be fulfilled in the Indonesian National Standard, SNI Portland Cement: 2049: 2015 [28].

There are four types of specimens, namely concrete with calcium stearate contents of 0, 0.1, 0.2, and 0.3% by weight of cement. Compressive strength specimens were only taken for the control specimen of concrete with 0% calcium stearate content. The chemical content tests, as shown in Table 3, showed that the OPC used contained 1.73% of MgO and 2.06% of SO_3_. The content of free lime, insoluble parts and alkalis are 1.53, 0.71 and 0.44%. Two test pieces are taken for each specimen code.

### 2.2. Procedure for Specimens Preparation and Testing

In this work, we conduct tests on the influence of the calcium stearate to the shrinkage property of concrete. The test is done with standard shrinkage test according to ASTM C 157/ C 157M [26]. Based on the code, the temperature room is controlled around 22.8 ± 1.7 °C, and relative humidity is around 50 ± 4%. The shrinkage of concrete specimen 100 × 100 × 285 mm is measured using automatic dial gauge with accuracy around 0.001 mm. The contents of calcium stearate investigated in this study are 0, 0.1, 0.2, and 0.3% by weight of cement. The number of specimen is two pieces for each calcium stearate content in concrete.

Fresh concrete is produced by mixing cement, crushed stone, sand, water, and calcium stearate together. The first step is to put the cement and sand into a concrete mixer and to mix it until it is uniformly mixed to form homogeneous material. The concrete mixer is then stopped and the crushed stone is added for the second mixing process. The last step is to add water according to the mix design. The concrete mixer is rotated again to form homogeneous fresh concrete.

Meanwhile, slump testing is performed to determine the workability of the fresh concrete. After the target of concrete workability is achieved, calcium stearate is added to the concrete mixer and the fresh concrete is rotated again. After the fresh concrete is quite mature and homogeneous, it is cast in the molding cylinder for the compressive strength specimens and into the beam with dimensions 100 × 100 × 285 mm for the shrinkage test.

**Figure 3:**
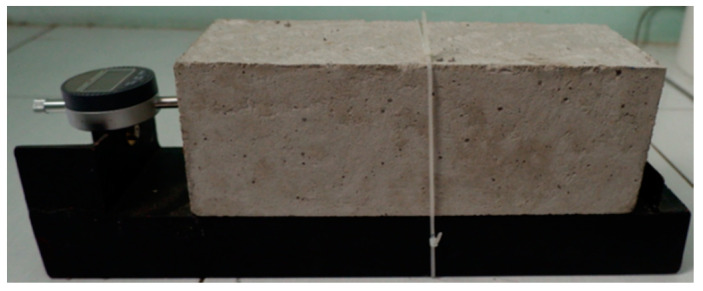
Set-up of digital micro indicator.

Cylinder and beam test specimens are treated by covering them with a wet mattress. Curing is carried out once the specimen is 1–2 h old and the fresh concrete gradually becomes hard. The test object is removed from the mold when it has reached 24 h of age. The dimensions of the shrinkage specimens are measured to determine the length and height at three different places. In addition to measuring the dimensions, the shrinkage specimen is also weighted with a digital scale. Figure 3 shows the set-up to measure the shrinkage of specimen using a digital micro indicator.

### 2.3. Shrinkage Modelling Approach

According to our knowledge, a direct influence of calcium stearate to existing standard shrinkage models has not been studied before. In this work, we use the Sakata [23], the Japan Standard [24] and the Bazant-Baweja [25] models to incorporate the influence of the calcium stearate to the standard concrete shrinkage model. We propose to use the models, which explicitly contain water content as a variable, because our initial prediction on the shrinkage behaviors due to the influence of calcium stearate is related to the water content ratio. It is important to note here that our modelling efforts are merely to investigate the possible connection between the calcium stearate and the shrinkage behaviors.

## 3. Results

### 3.1. Compressive Strength

Concrete compressive strength testing was conducted when the concrete was at the age of 28 days. The results of the compressive strength of the two test specimens of concrete with OPC as a binder are 21.94 and 21.50 MPa, corresponding to an average compressive strength of 21.22 MPa. This shows that the mix proportion designed has fulfilled the 20 MPa concrete compressive strength plan. It is worth noting here that the effect of calcium stearate on compressive strength of self-compacting concrete (SCC) has been studied in [21]. The result showed that the compressive strength of SCC at grade 20 MPa decreased around 7.5% due to the addition of calcium stearate 5 kg/m^3^ of concrete.

### 3.2. Density

The results of the measurement of the average length, width, height, and weight of each shrinkage test specimen are shown in Table 5.

In Table 5, SCS represents the specimens for shrinkage, while the numbers 0, 1, 2, and 3, represent the calcium stearate content added to the concrete: 0.0, 0.1, 0.2, and 0.3% of the weight of cement, respectively.

### 3.3. Shrinkage by Experiment

**Figure 4:**
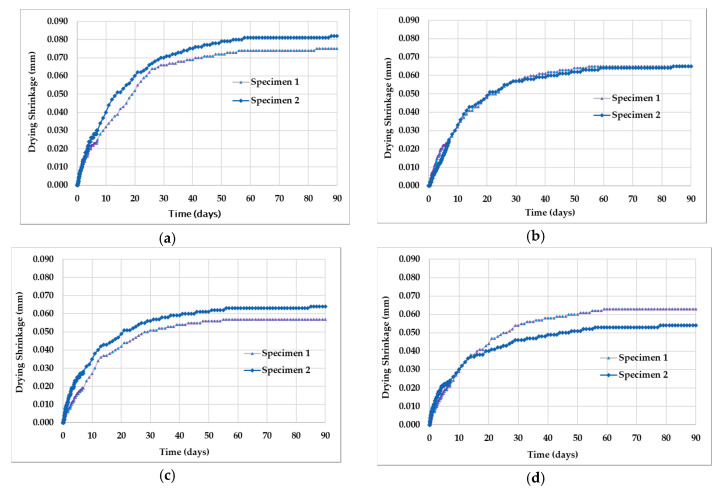
Drying shrinkage versus age of concrete specimen: (**a**) without calcium stearate (SCS0); (**b**) with calcium stearate 0.1% (SCS1); (**c**) with calcium stearate 0.2% (SCS2); (**d**) with calcium stearate 0.3% (SCS3).

**Figure 5:**
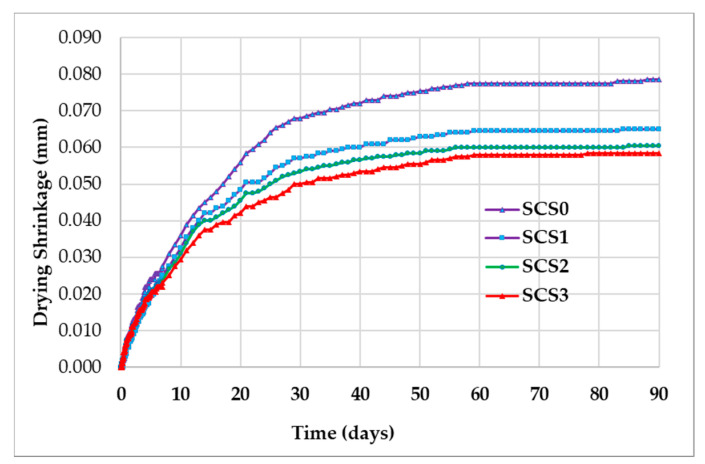
Average of drying shrinkage versus age of concrete from the results in Figure 4.

Figure 4 shows the amount of shrinkage on a concrete specimen measured for 90 days. Figure 4a–d represents drying shrinkage on the concrete specimen with calcium stearate 0, 0.1, 0.2, and 0.3% by weight of cement. Furthermore, Figure 5 shows the average amount of drying shrinkage for all fractions of calcium stearate.

**Figure 6:**
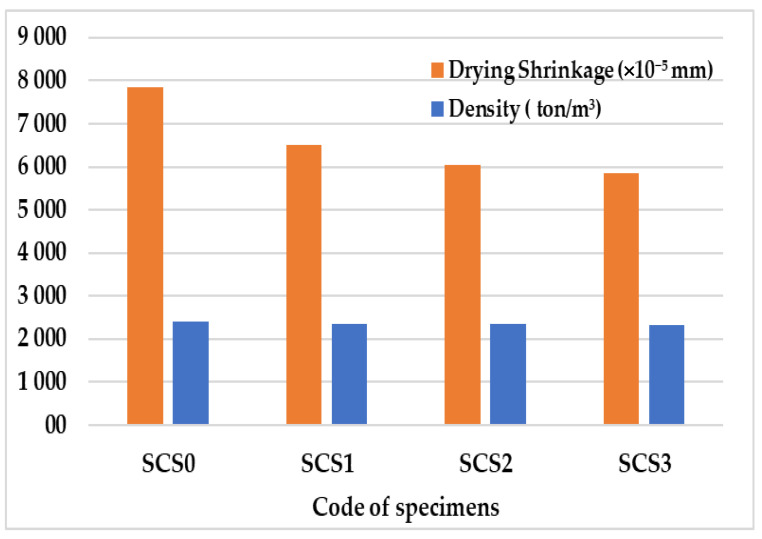
Drying Shrinkage and density of specimens.

Additionally, the density and drying shrinkage in the concrete at 90 days age are shown in Figure 6.

### 3.4. Shrinkage by Modelling

**Figure 7:**
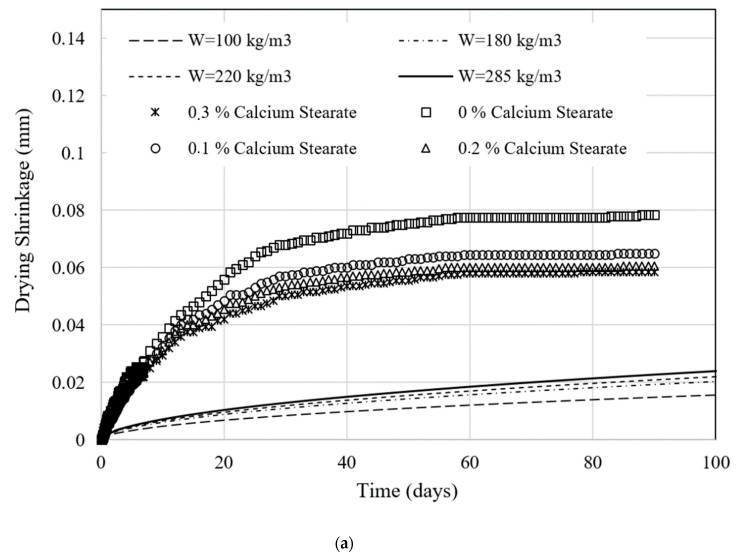

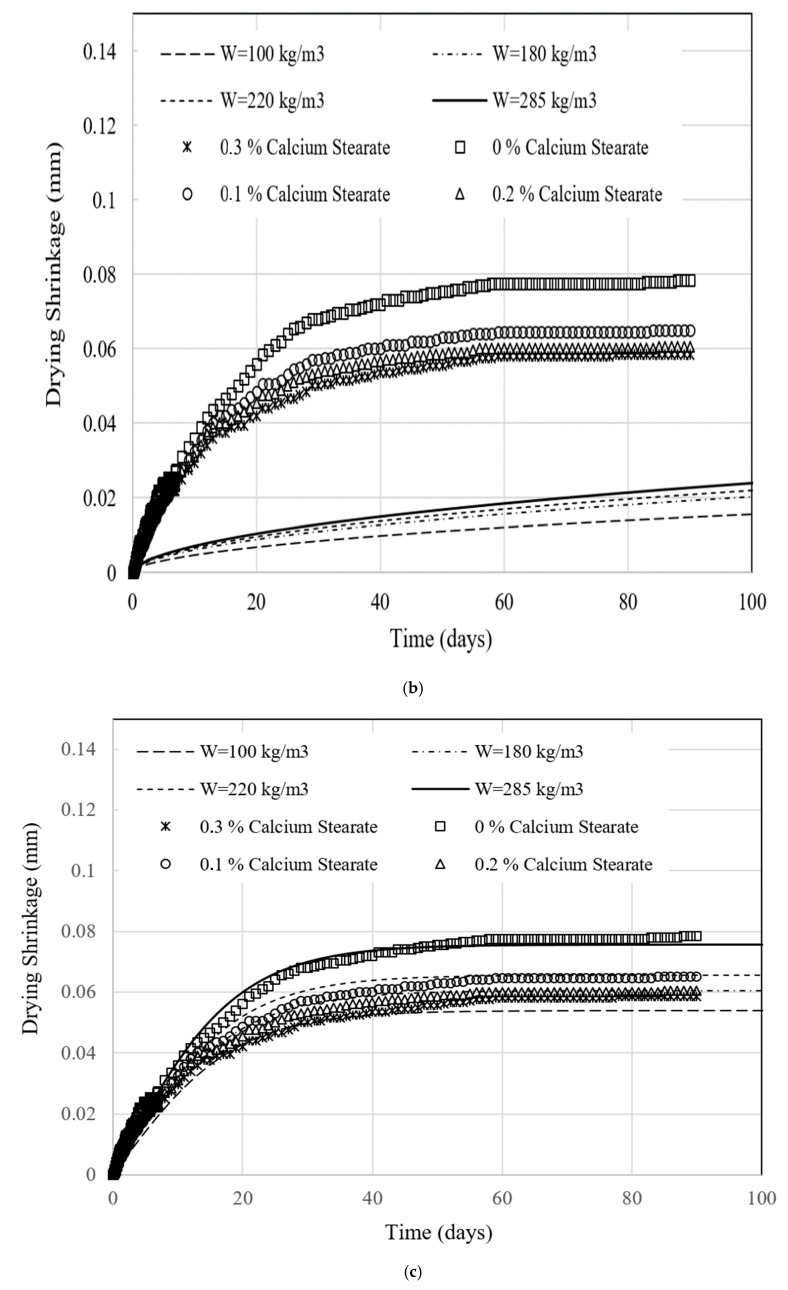
Comparison between the shrinkage model, i.e., (**a**) the Sakata [23], (**b**) the Japan Standards [24] and (**c**) the Bazant–Baweja [25] models with water content variations and the experiment with different fractions of calcium stearate.

The result of shrinkage value by experiment and models of the Sakata [23], the Japan Standard [24] and the Bazant–Baweja [25] are shown in Figure 7.

The results in Figure 7 are produced by fitting process with our shrinkage tests in Figure 5 for the same interval value of water content W. Here, we basically use Figure 6 to show two types of information simultaneously, i.e., the influence of calcium stearate from our tests and the influence of the water content from the modeling. By doing this, one can see the possible relation between the fraction of calcium stearate and the water content to the standard shrinkage model. The formulation of the standard shrinkage model can be rewritten as follows.

The Sakata model [23] is formulated with five main equations as follows.

(1) $$\varepsilon_{sh}\left(t,t_{0}\right)=\frac{\varepsilon_{sh,\infty }\left(t-t_{0}\right)}{\beta +\left(t-t_{0}\right)}$$

(2) $$\beta =\frac{4W\sqrt{V/S}}{100+0.7t_{0}}$$

(3) $$\varepsilon_{sh,\infty }=\frac{\varepsilon_{sh,\rho }}{1+\eta t_{0}}$$

(4) $$\varepsilon_{sh,\rho }=\frac{\alpha \left(1-h\right)W}{1+150\;\exp\left(-\frac{500}{f_{c}^{\prime }\left(28\right)}\right)}$$

(5) $$\eta =10^{-4}\left[15\;\exp\left(0.007\;f_{c}^{\prime }(28)\right)+0.25W\right]$$

The Japan Standards model [24] is formulated with two main equations as follows.

(6) $$\varepsilon_{sh}\left(t,t_{0}\right)=\left(1-e^{-0.018{\left(t-t_{0}\right)}^{0.56}}\right)\varepsilon_{sh,\infty }$$

(7) $$\varepsilon_{sh,\infty }=-50+78\left[1-\exp\left(\frac{RH}{100}\right)\right]+38\;\ln W-5{\left[\ln\left(\frac{V/S}{10}\right)\right]}^{2}$$

The Bazant–Baweja model [25] is formulated with two main equations as follows.

(8) $$\varepsilon_{sh}\left(t,t_{0}\right)=-\varepsilon_{sh,\infty }k_{h}\tanh\left(\frac{t-t_{0}}{\tau_{sh}}\right)$$

(9) $$\varepsilon_{sh,\infty }=-\alpha_{1}\alpha_{2}\left[0.00856\;W^{2.1}\;{\left(145f_{c}^{\prime }\right)}^{-0.28}+270\right]\frac{\sqrt{\frac{607}{4+0.85\times 607}}}{\sqrt{\frac{t_{0}+\tau_{sh}}{4+0.85(t_{0}+\tau_{sh})}}}$$

As we assume that the correlation between the fraction of calcium stearate and the water content W exists, shrinkage behaviors from the models must be plotted first. Then, with respect to the change of W, the remaining variables in Table 6 are kept constant. Finally, we insert the shrinkage test results from Figure 5 as the reference curves to find the constants value listed in Table 6.

## 4. Discussion

Based on the mix proportion of concete in Table 4, we show that the variable of the mixed design of concrete applied here is calcium stearate fraction. There are four mix designs of concrete, which are concrete with 0, 0.1, 0.2, and 0.3% by weight of cement of calcium stearate content. The density and shrinkage of concrete with calcium stearate is compared to concrete without calcium stearate with the same controlled parameters in Table 4. Hence, any possible differences in physical and mechanical properties of concrete in the testing will be produced only by different fractions of calcium stearate.

Table 5 shows that the concrete density decreases as the addition of calcium stearate increases. This is most probably caused by a stearate group that reacts with calcium silicate hydrate (C-S-H) to form a wax-like compound. This compound is less dense and more hydrophobic compared to tobermorite (C-S-H). However, this reduction is less apparent than the shrinkage reduction as shown in Figure 6. Hence, we may conclude that the addition of calcium stearate is more influential to the change of shrinkage behaviors than the density of concrete. This is an interesting phenomena that is open to more investigations and tests.

Based on Figure 4 and Figure 5, it can be observed that the addition of calcium stearate to concrete can reduce shrinkage. The concrete shrinkage resulting from the addition of 0, 0.1, 0.2, and 0.3% calcium stearate to the weight of cement at 28 days is 0.067, 0.053, 0.056, and 0.049 mm, and at 90 days is 0.079, 0.065, 0.061, and 0.059 mm. This reduction is quite significant when associated with the possibility of cracking in concrete due to drying shrinkage. As previously explained, calcium stearate reacts with cement and water to form wax-like compounds that are hydrophobic. This shows that the wax-like compound has a more stable volume compared to tobermorite when the hydration process occurs in the cement. The water in the concrete evaporates and continues to decrease with the increasing age of the concrete. However, evaporation of water during the hydration process does not affect the volume of the compounds that resemble wax.

Recalling again Figure 6, there is still a possibility to explain a relationship between the slight change of density and the drying shrinkage reduction. The less dense the concrete, the smaller the drying shrinkage. This phenomenon can be explained by the fact that the reaction between calcium stearate with cement and water in concrete forms lighter compounds, and a more stable volume during the hydration process occurs in the concrete. The reaction result is shown below based on the ACI report [29].

2C3S + 6H → C3S2H3 + 3Ca(OH)_2_ (10)

Calcium silicate hydrate

Ca(OH)_2_ + RCOOH → Ca^+^COOR^−^ + H_2_O (11)

Calcium hydroxide(lime) + stearate → insoluble calcium stearate + water

**Figure 8:**
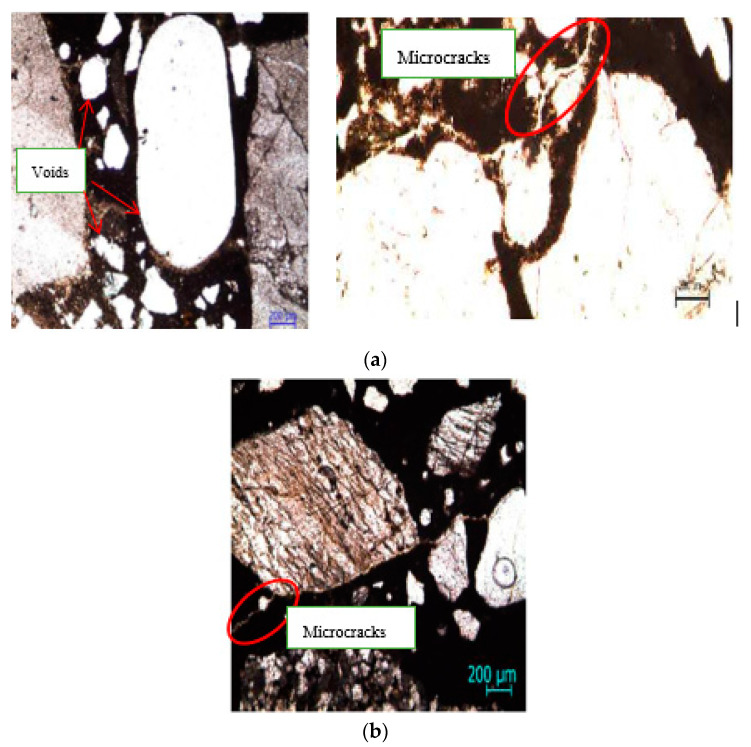
Photomicrograph of concrete [15]: (**a**) without calcium stearate; (**b**) with calcium stearate.

The smaller shrinkage of concrete with calcium stearate is also supported by Quraishi et. al. [15] research. The photomicrograph concrete without and with calcium stearate are shown in Figure 8. Figure 8b shows that calcium stearate fills in the smaller void and microcracks in the concrete. As the result, total void and microcracks in the concrete are reduced. More over, the shrinkage of concrete with calcium stearate decreases significantly (Figure 8b). A contrasting result is shown in Figure 8a (right) for concrete without calcium stearate, as some microcracks still appeared. On the other hand, Figure 8a (left) indicates the existence of many small to large voids in the concrete without calcium stearate.

Meanwhile, from the shrinkage modeling, we find that the cement shrinkage can be proportional to the water content in the concrete as shown in Figure 7. This finding is particularly very clear in the case of using the Bazant–Baweja model. The larger water content relates to the faster shrinkage rate. Interestingly, this behavior is similar to the influence of calcium stearate on the tests but in the opposite way where a higher concentration of calcium stearate produces slower shrinkage.

The shrinkage of concrete has the same trend both by experiment and modeling due to the addition of calcium stearate. It shows that the Bazan–Baweja model can predict experimental results on the shrinkage of concrete with calcium stearate very well. The next study should investigate the shrinkage of concrete with a binder of Ordinary Portland Cement and fly ash. It will be interesting because the setting time of concrete is usually slower due to the addition of fly ash in concrete.

## 5. Conclusions

Calcium stearate, Ca(C_18_H_35_O_2_)_2_, reacts with cement and water to form a hydrophobic layer on the surface of pores and promotes a wax-like (Ca^+^COOR^−^) microstructure. The compound has a lighter and more stable volume compared to calcium silicate hydrate/tobermorite (C-S-H). The reaction influences cement shrinkage behavior, which depends on the fraction of calcium stearate with respect to the cement weight.

Our experimental works show that the higher concentration of calcium stearate produces a smaller value of concrete shrinkage. It means that the additional calcium stearate in concrete reduces the shrinkage as well.

Finally, from the shrinkage modelling, we find that the cement shrinkage can be proportional to the water content in the concrete. This finding is particularly very clear in the case of using the Bazant–Baweja model. Because of this, the Bazant–Baweja model can be applied to predict the shrinkage of concrete with calcium stearate at any dosages and age of concrete.

## Figures and Tables

**Table 1:** Physical properties of sand and crushed stone.

Physical Properties	Crushed Stone	Sand
Specific gravity	2.55 (PBI 1971 [27] = no requirement)	2.65 (PBI 1971 = no requirement)
Density (ton/m^3^)	1.53 (PBI 1971 = no requirement)	1.65 (PBI 1971 = no requirement)
Fineness modulus	6.86 (PBI 1971 = no requrement)	2.67 (PBI 1971 = no requrement)
Clay content (%)	0.94 (PBI 1971 = max. 1%)	2 (PBI 1971 = max. 5%)
Abrasion with 500 rotation (%)	11 (PBI 1971 = max. 22%)	Not required (PBI 1971 = no requirement)

**Table 2:** Physical characteristics of ordinary Portland cement (OPC.

Physical Characteristic	Result	SNI Portland Cement 2049:2015 (OPC)
Fineness with Blaine apparatus (m^2^/kg)	360	Min. 280
Fineness with mesh 45 µ, residue (%)	10.34	-
Initial setting time (minutes)	137	Min. 45
Final setting time (minutes)	248	Max. 375
Expansion	0.04	Max. 0.80
Compressive strength (kg/cm^2^)		
−3 days	228	Min. 135
−7 days	284	Min. 215
−28 days	390	Min. 300
False set	88.78	Min. 50

**Table 3:** Chemical content of OPC.

Chemical Content	OPC (%)	SNI Portland Cement 2049:2015 for OPC
Silicon oxide (SiO_2_)	18.76	-
Alumunium oxide (Al_2_O_3_)	5.54	-
Ferric oxide (Fe_2_O_3_)	3.31	-
Calcium oxide (CaO)	63.16	-
Magnesium oxide (MgO)	1.73	Max. 6.00
Sulphur trioxide (SO_3_)	2.06	Max. 3.50
Loss of ignition	3.73	Max. 5.00
Free lime	1.53	-
Insoluble part	0.71	Max. 3.00
Alkali (Na_2_O + 0.658 K_2_O)	0.44	Max. 0.60

**Table 4:** Mix proportions of concrete.

Materials	Weight (kg)	Volume (m^3^)
Cement (OPC)	324	0.114
Crushed stone	940	0.376
Sand	805	0.292
Water	205	0.205
Calcium stearate (%)	0; 0.1; 0.2; 0,3	≈0.00
Water cement ratio	0.63	-
Sand/aggregate	0.46	-
Void	-	0.015
Total		1.002

**Table 5:** Average dimensions and weight of the shrinkage specimens.

Code	Number of Specimen	Length (mm)	Width (mm)	Thickness (mm)	Volume (mm^3^)	Weight (g)	Density (g/cm^3^)
SCS0	1	100.05	100.07	285.08	2,854,222	6898	2.42
	2	99.05	98.01	283.15	2,748,789	6589	2.40
SCS1	1	100.14	100.22	285.33	2,863,580	6801	2.37
	2	100.03	100.01	285.17	2,852,840	6709	2.35
SCS2	1	100.04	100.03	285.12	2,853,196	6701	2.35
	2	100.87	100.75	285.93	2,905,807	6808	2.34
SCS3	1	100.95	100.88	285.97	2,912,271	6800	2.33
	2	100.42	100.51	285.71	2,883,732	6702	2.32

**Table 6:** Material constants for the model [23,24,25].

Material Constant	Description	Values in Figure 6		
		**[24]**	**[25]**	**[26]**
W	Water content [kg/m^3^]	varied	varied	varied
V/S	Volume-surface ratio [mm]	275	7.0	-
R/H	Relative humidity [no unit]	-	50	-
$k_{h}$	Humidity-dependent factor [mm]	-	-	1.0
$f_{c}^{\prime }$	Concrete compressive strength at 28 days [MPa]	21	-	21
h	Relative humidity [no unit]	0.45	-	-
α	Cement type factor [no unit]	18.0	-	-
$\alpha_{1}$	Cement type factor [no unit]	-	-	1.7
$\alpha_{2}$	Curing factor [no unit]	-	-	1.0
$\tau_{sh}$	Size and shape-dependent factor [days]	-	-	18.5
$t_{0}$	Initial time [days]	0	0	0
t	Shrinkage time [days]	from test	from test	from test
